# Overweight among students aged 11–15 years and its relationship with breakfast, area of residence and parents’ education: results from the Italian HBSC 2010 cross-sectional study

**DOI:** 10.1186/1475-2891-13-69

**Published:** 2014-07-05

**Authors:** Giacomo Lazzeri, Mariano Vincenzo Giacchi, Angela Spinelli, Andrea Pammolli, Paola Dalmasso, Paola Nardone, Anna Lamberti, Franco Cavallo

**Affiliations:** 1 CREPS - Center of Research for Health Education and Promotion, Department Molecular and Developmental Medicine, University of Siena, Via A. Moro 2, 53100 Siena, Italy; 2 National Center for Epidemiology, Surveillance and Health Promotion, National Institute of Health, Roma, Italy; 3 Department of Public Health and Pediatrics, University of Torino, Torino, Italy

**Keywords:** Adolescents, BMI, Breakfast, Education, Residence

## Abstract

**Background:**

The international increase in overweight and obesity among children and adolescents over the past three decades confirms that childhood obesity is a global ‘epidemic’. The World Health Organization considers childhood obesity to be a major public health concern. Childhood obesity is associated with cardiovascular, endocrine, musculoskeletal and gastrointestinal complications, and may have psycho-social consequences. The aim of this paper is to examine overweight (including obesity) prevalence and its association with geographic area of residence, parental education and daily breakfast consumption in Italian students aged 11–15 yrs.

**Methods:**

A nationally representative sample of 11–15 year old students from 20 Italian Regions (Italian Health Behaviour in School-aged Children 2010-HBSC) was randomly selected (2,504 schools and 77,113 students). Self-reported anonymous questionnaires, prepared by the international HBSC network, were used to collect the data. BMI was calculated using self-reported weight and height and the International Obesity Task Force cut-offs. Multiple logistic regressions were performed to assess the relationship between the risk of overweight and parental education, area of residence and breakfast consumption in each age group and gender.

**Results:**

Boys were more likely to be overweight or obese than girls (28.1% vs. 18.9% at 11 yrs-old, 24.8% vs. 16.5% at 13 yrs and 25.4 vs. 11.8% at 15 yrs). The prevalence of overweight and obesity was lower among the older girls. Overweight and obesity rates increased from the North of Italy to the South in both boys and girls and in all age groups. Boys 11-15 yrs living in southern Italy had an OR=2.05 (1.77-2.38) and girls 2.04 (95% CI 1.70-2.44) for overweight (including obesity) compared with those living in the North. Parent’s low educational level and no daily breakfast consumption were also associated with overweight including obesity (p<0.05).

**Conclusion:**

The prevalence of obesity and overweight in Italian school-children 11-15 yrs old are high, in particular in the South and in boys. These findings suggest appropriate interventions are needed, at the community as well as the individual level, in particular in the southern regions. However, more research is warranted on intermediary factors to determine which interventions are likely to be most effective.

## Background

The increase in overweight and obesity among children and adolescents in both developed and developing countries over the past three decades confirm that childhood obesity is a global ‘epidemic’ [1-4]. The World Health Organization (WHO) considers childhood obesity to be a major public health concern [5]. Childhood obesity is associated with cardiovascular, endocrine, pulmonary, musculoskeletal and gastrointestinal complications, and may have psycho-social consequences (poor self esteem, depression, eating disorders) [6].

One of the most important nutritional factors is breakfast, the first of the three main meals of the day [7]. Skipping breakfast has been associated with increased probability of being overweight both in children and adults [8,9]. Prospective studies have confirmed this association in adolescents [10].

Obesity is a multi-factorial disorder originating from the interaction between genetics and environment [11-13]. The accumulation of body fat is a very complex phenomenon regulated by a series of physiological mechanisms, some of which are still unknown. Family lifestyles have a big impact on the nutritional and behavioural choices of children, together with social and economic factors, such as place of residence, parental educational level and economic affluence [14]. It is well known that there is an inverse relationship between socio-economic conditions and health status in developed countries [15]. Specifically, studies have also shown an inverse relationship between children’s Body Mass Index (BMI) and family educational level [14,16].

Recent data comparing the Italian national data with the other 13 European countries participating in the WHO Europe Childhood Obesity Surveillance Initiative (COSI) show that Italy had the highest prevalence of overweight and obesity among children aged 8–9 [17]. Moreover, several surveys conducted at local or regional level, in Italy, have found geographic differences in pediatric obesity prevalence [18-20], although its magnitude has been difficult to evaluate because of the differences in methods and definitions, and the limited geographic coverage. The only study that presents estimates of overweight and obesity prevalence in Italian primary school children by regions is “OKkio alla SALUTE”, a national surveillance system in which more than 45,000 third-grade (8–9 year old) students are measured every two years [21-23]. These data show a clear geographic trend of overweight and obesity prevalence increasing from North to South and the influence of demographic characteristics. In 2010, for the first time in Italy, the Health Behaviour in School-aged Children (HBSC) study was conducted on regionally representative samples. This paper presents the results on the prevalence of overweight and obesity in 58,928 Italian children (aged 11,13, and 15 years) by geographic area of residence. It also evaluates the association between childhood and adolescent overweight (including obesity) and gender, parental education and breakfast consumption. These results can be compared with those obtained for children aged 8–9 years obtained from “OKkio alla SALUTE” surveillance. It is important to get information at these older ages because most of these adolescents have already had their pubertal development and are more independent in their nutritional and physical activity choices.

## Methods

Data were collected in accordance with the HBSC international protocol developed and regularly updated by the research group, with the participation of research workers from each member state. HBSC is a WHO collaborative cross-sectional study, involving research teams across Europe and North America, with the aim of gaining insight into adolescents’ health and health behaviour. It collects data every four years on 11, 13 and 15-year-old boys’ and girls’ health and well-being, social environment and health behavior. The protocol describes the methods for performing the survey, the rules to be followed and coding procedures for the data collected [24,25]. All participating countries must adhere to the detailed version of the protocol. In Italy, in contrast with the two previous rounds of HBSC, all regions decided to have their own representative sample to allow comparisons at regional level.

### Sampling

According to the rules agreed internationally, one-stage cluster sampling was used with classes within schools as primary sampling unit. Schools and classes were stratified by region and in each of them by grade (middle and high schools) [26,27]. The selection of the classes was made using sampling with probability proportional to size and the sample size for each region was about 1,200 children for each age 11, 13 and 15 years, corrected for the general population of students. Over-sampling from 10% to 25% was applied in each age group to compensate for the differences in the children’s ages and for those expected to refuse to participate.

### Data collection

Data collection began in late November 2009 and lasted until the end of May 2010. All Italian regions were involved, but Veneto carried out the survey independently and Piedmont, which carried out the survey in 2009, in agreement with the coordination group, did not deem it necessary to repeat it after such a short time. To collect data, a self-reported anonymous questionnaire, prepared by the international HBSC network, was used. Data were collected by trained health workers in collaboration with the school teachers. Further details of the methodology of the Italian HBSC 2010 study are provided elsewhere [27].

### Variables

Variables included in the analyses were: children’s age, gender, weight, height, region of residence, breakfast consumption and parental educational level.

Children’s BMI (kg/m^2^) was calculated using self-reported weight and height, and body weight status was assessed according to Cole’s classification, as recommended by the International Obesity Task Force [28,29], in four categories: underweight (U), normal-weight (N), overweight (Ow) and obese (O). In these analyses, we have also considered overweight including obesity (OwO).

Regions of residence were grouped into northern, central and southern areas using the Italian National Statistics Institute classification [30].

On the basis of the strong correlation between educational status and income in Italy [31], the higher educational level of the mother or father was used as a proxy of socio-economic status. Responses to the question on education were grouped: less than high school, high school, university degree or more.

To assess breakfast consumption, adolescents were asked to indicate, in a normal week, how many days in weekdays and in weekends they had breakfast (defined as having more than a glass of milk or fruit juice). Response categories were “never” to “five days” for the weekdays, and “never” to “two days” for the weekend. The number of weekdays and weekend days were summed and dichotomized into “daily breakfast consumption” (seven days in a week) vs. “less than daily” (less than seven days in a week).

### Data analysis

A central automatic data entry system was used. Cases were deleted if gender and/or age were missing. Exclusion criteria in the analyses were age outside the range of ± 6 months compared to the average age of its own stratum, and abnormal values for weight and/or height (5 Kg below 3^rd^ or 30 Kg above the 97^th^ percentile; 5 cm below 3^rd^ or 5 cm above the 97^th^ percentile) [24]. Overweight and obesity prevalence rates and 95% Confidence Intervals (CI) were estimated separately by age and gender [24]. Multiple logistic regressions were performed to assess the relationship between overweight (including obesity) as the dependent variable, and parental education, area of residence and breakfast consumption as independent variables (each model was adjusted simultaneously for parental education, breakfast consumption and children’s residence area). All analyses were conducted taking into account the survey design (including stratification, clustering and weighting) using STATA 12.1 SE Surveys routines.

### Ethical aspects

Parents had to consent to the participation of their children in the HBSC survey. The Ethics Committee of the National Institute of Health, which approved the protocol and instruments of the Italian HBSC 2010 study, agreed to the use of an opt-out consent form, in which parents were asked to explicitly refuse permission to participate and the lack of a returned form was taken to imply consent. Since the HBSC survey aims to collect data relating to the population and avoids the identification of individuals, the students answered the questionnaires anonymously. As per protocol, questionnaires, once completed, were collected and immediately placed in a sealed envelope by a health workers. All information collected cannot be traced to the individual student.

## Results

Overall, the Italian HBSC 2010 survey included 2,504 schools and 77,113 students. After data screening and applying the inclusion criteria, over 58,000 students (76.4% of the total) were considered eligible for the analysis. Table 1 shows the main characteristics of the sample. Each age group represented almost one-third of the sample with a 1:1 male:female ratio. Almost half of them were resident in the South (49.6%), a third in the North (33.8%) and 16.6% in Central Italy. Regarding parents’ education, 28% of students had at least one parent with a University degree. On average boys were younger, tend to be resident in the north and have more educated parents. Less than 50% of 11-15 yrs school-children had breakfast every day; in particular girls had a lower percentage than boys (44.1% vs. 51.4%). The overall combined prevalence of overweight and obesity was 20.9% according to IOTF criteria, while the prevalence of obesity alone was 3.4%, with higher values among boys (Ow: 21.5% and O: 4.8% vs. Ow: 13.6% and O: 2.0%among girls).

**Table 1:** Socio-demographic characteristics of study participants by gender

		**Boys**		**Girls**		**All**	
		**N**	**%**	**N**	**%**	**N**	**%**
**Age**	**11 y**	10600	37.4	10128	34.1	20728	35.7
	**13 y**	10244	34.0	10417	35.2	20661	34.6
	**15 y**	8459	28.6	9080	30.7	17539	29.7
	**Total**	29303	100	29625	100	58928	100
**Geographicarea of residence**	**North**	11823	34.2	11954	33.4	23777	33.8
	**Centre**	6451	17.4	6110	15.8	12561	16.6
	**South**	11029	48.4	11561	50.8	22590	49.6
	**Total**	29303	100	29625	100	58928	100
**Parental education attainment°**	**Less than high school**	7169	33.1	8264	38.0	15433	35.6
	**High school diploma**	8906	36.4	9038	36.5	17944	36.4
	**University degree**	7305	30.5	6472	25.5	13777	28.0
	**Total**	23380	100	23774	100	47154	100
	*Missing*	*5923*	*20.6*	*5851*	*19.0*	*11774*	*19.8*
	**Daily**	15487	51.4	13707	44.1	29194	47.8
**Breakfast**	**Less than daily**	13603	48.6	15717	55.9	29320	52.2
	**Total**	29090	100.0	29424	100.0	58514	100.0
	*Missing*	*213*	*0.7*	*201*	*0.6*	*414*	*0.6*
	**Underweight**	403	1.7	796	3.0	1199	2.4
	**Normal-weight**	17715	72.1	20200	81.4	37915	76.7
**BMI**	**Overweight**	4337	21.5	2857	13.6	7194	17.5
	**Obese**	849	4.8	426	2.0	1275	3.4
	**Total**	23304	100.0	24279	100.0	47583	100.0
	*Missing*	*5999*	*20.9*	*5346*	*19.2*	*11345*	*20.0*

° Higher educational level between mother and father.

**Figure 1:**
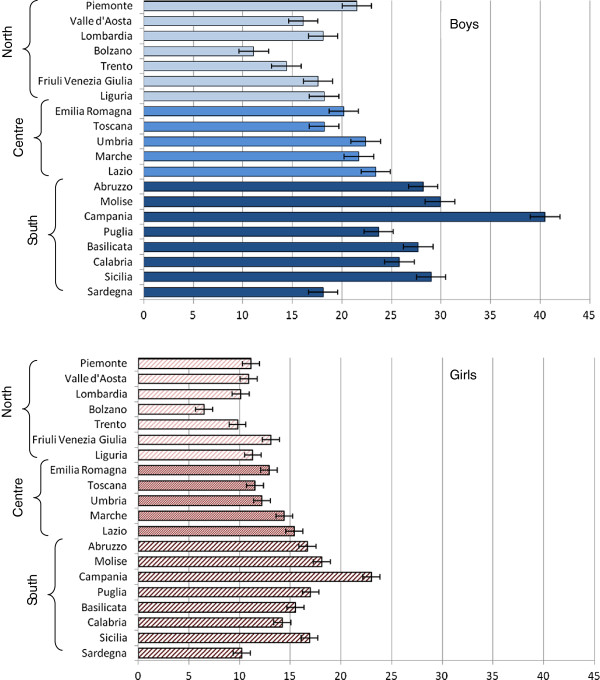
Prevalence of OwO in the Italian regions by gender.

Table 2 presents weight status prevalence rates by age, gender and geographical area. At every age and in all geographical areas, boys were more likely to be overweight (including obesity). Overall 28.1% of boys and 18.8% of girls aged 11 yrs-old, 24.8% and 16.5% aged 13 yrs, and 25.4% vs. 11.7% aged 15 yrs were overweight or obese. A geographical trend was found in both boys and girls and in all age groups: overweight and obesity rates increase from North to South. Among girls a lower percentage was overweight (including obesity) in the group of 15-year olds (11.7%, 95% CI: 10.6%-13.4%) compared both with the group aged 13 years (16.5%, 95% CI: 15.0%-18.3%) and 11 years (18.8%, 95% CI: 17.1%-20.7%), whereas boys showed the lowest prevalence at thirteen years (24.8%, 95% CI: 22.8%-27.0%), an intermediate prevalence at 15 years (25.4%, 95% CI: 23.1%-27.4%) and then the highest prevalence in youngest, 11 years old (28.1%, 95% CI: 26.0%-30.2%).The lowest value of overweight including obesity in Italy in 2010 was found in the Northern 15 yrs-old girls (7.1%, 95%CI:5.5%-8.7%) and the highest in the Southern 11 yrs-old boys (36.0%, 95%CI: 33.2%-39.4%). Overall the regions with highest and lowest prevalence were Campania and the Province of Bolzano (Figure 1).

**Table 2:** Prevalence of underweight (U), normal-weight (N), overweight (Ow) and obesity (O) by students’ age, gender and residence area, Italy, 2010

		**Boys**				**Girls**			
**Age**		**U**	**N**	**OW**	**O**	**U**	**N**	**OW**	**O**
		**% (95% CI)**	**% (95% CI)**	**% (95% CI)**	**% (95% CI)**	**% (95% CI)**	**% (95% CI)**	**% (95% CI)**	**% (95% CI)**
**11 y**									
	**North**	1.9 (1.17-2.91)	79.3 (76.60-81.77)	14.5 (12.65-16.65)	4.3 (3.17-5.85)	3.3 (2.23-4.72)	83.5 (80.72-85.92)	11.6 (9.39-14.35)	1.6 (1.00-2.62)
	**Center**	2.3 (1.47-3.72)	72.4 (68.50-75.95)	21.3 (18.41-24.60)	3.9 (2.76-5.59)	3.9 (2.44-6.13)	80.2 (76.65-83.36)	13.5 (10.84-16.72)	2.4 (1.46-3.84)
	**South**	2.5 (1.78-3.44)	61.5 (58.53-64.43)	28.5 (25.54-31.68)	7.5 (6.01-9.28)	3.4 (2.50-4.65)	72.6 (69.75-75.31)	21.1 (18.66-23.76)	2.9 (2.01-4.07)
	**Total**	2.2 (1.75-2.82)	69.7 (67.58-71.76)	22.3 (20.41-24.24)	5.8 (4.90-6.85)	3.4 (2.76-4.24)	77.7 (75.85-79.42)	16.5 (14.94-18.28)	2.3 (1.82-3.02)
**13 y**									
	**North**	1.5 (0.88-2.60)	80.0 (77.17-82.59)	15.4 (13.24-17.90)	3.0 (2.11-4.35)	2.9 (2.00-4.21)	85.3 (82.90-87.32)	10.1 (8.49-12.01)	1.7 (0.98-3.05)
	**Center**	1.2 (0.62-2.41)	79.8 (75.84-83.19)	16.2 (13.32-19.51)	2.8 (1.87-4.28)	3.8 (2.52-5.57)	83.0 (79.84-85.66)	12.2 (9.88-15.05)	1.1 (0.53-2.10)
	**South**	1.7 (1.13-2.67)	67.0 (63.42-70.30)	25.7 (22.70-28.90)	5.6 (3.77-8.32)	2.1 (1.40-3.13)	77.5 (74.80-80.06)	17.2 (14.95-19.72)	3.2 (2.25-4.42)
	**Total**	1.6 (1.16-2.15)	73.6 (71.39-75.76)	20.5 (18.70-22.48)	4.3 (3.23-5.62)	2.6 (2.05-3.29)	80.9 (79.16-82.49)	14.1 (12.71-15.68)	2.4 (1.81-3.15)
**15 y**									
	**North**	1.8 (0.96-3.17)	79.4 (76.58-82.03)	16.3 (13.78-19.25)	2.5 (1.60-3.82)	3.5 (2.44-4.86)	89.5 (87.48-91.22)	6.4 (5.07-7.95)	0.7 (0.36-1.33)
	**Center**	1.6 (0.85-2.81)	78.4 (75.34-81.25)	18.3 (15.48-21.56)	1.7 (0.94-3.00)	3.6 (2.46-5.32)	82.9 (79.44-85.79)	12.6 (9.83-16.02)	0.9 (0.44-1.90)
	**South**	1.0 (0.58-1.73)	67.4 (63.60-71.04)	25.9 (22.62-29.56)	5.6 (4.20-7.50)	2.9 (2.14-3.89)	83.2 (80.97-85.18)	12.1 (10.08-14.44)	1.8 (1.21-2.77)
	**Total**	1.3 (0.95-1.89)	73.3 (71.02-75.40)	21.5 (19.51-23.62)	3.9 (3.08-4.93)	3.2 (2.61-3.88)	85.1 (83.60-86.40)	10.4 (9.14-11.88)	1.3 (0.95-1.86)

The importance of the area of residence is confirmed in the multiple logistic regression models (Table 3). Children living in southern Italy showed a significantly doubled risk of overweight (OwO) compared with those living in North (OR = 2.05, CI 1.77-2.38 in boys and OR = 2.04, 95% CI 1.70-2.44 in girls). Overweight (OwO) and parent’s educational level were inversely associated: students with both parents in the lower educational level are more likely to be overweight than those with at least one parent with the highest educational level (OR = 1.63, 95% CI: 1.38-1.91 in boys; OR = 2.07, 95%CI: 1.70-2.51 in girls). This relationship is significant and consistent across gender and age groups (Table 3). Among all age groups and both in girls and boys, less than daily breakfast consumption was found to be associated with overweight (OwO) (OR = 1.33, 95% CI: 1.16-1.51 in boys; OR = 1.58, 95% CI: 1.38-1.82 in girls). Because nearly 20% of replies on educational level were missing, multiple regression analyses were also performed stratified by age and gender including the non-responders recoded as “other” category. The results of these analyses showed the same overall conclusions as main analyses, but with slightly attenuated associations (data not shown).

**Table 3:** Association of parental education, students’ residence area and breakfast consumption with overweight (OwO) – (multiple logistic regression), Italy, 2010

	**Prevalence of overweight including obesity (OwO)**															
	**11 y**				**13 y**				**15 y**				**All age group**			
	**Boys**		**Girls**		**Boys**		**Girls**		**Boys**		**Girls**		**Boys**		**Girls**	
	**OwO%**	**OR (95% CI)**	**OwO%**	**OR (95% CI)**	**OwO%**	**OR (95% CI)**	**OwO%**	**OR (95% CI)**	**OwO%**	**OR (95% CI)**	**OwO%**	**OR (95% CI)**	**OwO%**	**OR (95% CI)**	**OwO%**	**OR (95% CI)**
**Parental educational level**																
Less than high school	34.4	1.52* (1.18-1.96)	24.3	2.05* (1.47-2.85)	28.6	1.62* (1.19-2.20)	21.7	2.02* (1.44-2.82)	32.0	1.82* (1.37-2.42)	16.7	2.17* (1.54-3.08)	31.5	1.63* (1.38-1.91)	20.4	2.07* (1.70-2.51)
High school	26.8	1.16 (0.91-1.48)	18.5	1.54* (1.09-2.16)	25.3	1.48* (1.11-1.97)	15.7	1.46* (1.02-2.09)	24.4	1.28 (1.00-1.64)	10.6	1.34 (0.94-1.92)	25.3	1.27* (1.10-1.48)	14.2	1.39* (1.14-1.70)
University	22.9	Referent group	12.5	Referent group	18.3	Referent group	11.3	Referent group	19.8	Referent group	7.9	Referent group	20.5	Referent group	10.4	Referent group
**Residence area**																
North	18.9	Referent group	13.3	Referent group	18.5	Referent group	11.9	Referent group	18.8	Referent group	7.1	Referent group	18.7	Referent group	10.8	Referent group
Centre	25.8	1.68* (1.20-2.35)	16.3	1.56* (1.09-2.22)	20.3	1.25 (0.91-1.71)	13.7	1.43* (1.01-2.03)	20.3	1.26 (0.97-1.63)	13.8	2.45* (1.73-3.47)	22.1	1.35* (1.14-1.61)	14.5	1.73* (1.40-2.13)
South	36.2	2.49* (1.91-3.24)	24.1	2.07* (1.52-2.82)	31.2	1.77* (1.37-2.27)	20.4	2.03* (1.51-2.73)	31.3	2.02* (1.57-2.58)	14.1	2.08* (1.54-2.80)	33.0	2.05* (1.77-2.38)	19.2	2.04* (1.70-2.44)
**Breakfast consumption**																
Daily	24.9	Referent group	13.5	Referent group	22.0	Referent group	13.1	Referent group	21.5	Referent group	8.4	Referent group	23.0	Referent group	12.7	Referent group
Less than daily	32.1	1.33* (1.07-1.65)	22.8	1.88* (1.44-2.44)	28.0	1.29* (1.01-1.63)	19.3	1.61* (1.28-2.01)	28.5	1.41* (1.11-1.77)	13.9	1.71* (1.34-2.19)	29.5	1.33* (1.16-1.51)	18.0	1.58* (1.38-1.82)

Odd ratios and 95% CIs were calculated from logistic regression models stratified by age and gender. Each model was adjusted simultaneously for parental education, breakfast consumption and students’ residence area.

*p < 0.05.

## Discussion

This study, which used standardized methods and equipment, is the only recent population-based investigation of BMI in 11-15 years old students in Italy where it is possible to make comparisons between regions*.* In addition to a high prevalence of overweight and obesity in the overall population, which is higher than that of most Western countries [25], there were substantial geographic differences, with the prevalence of obesity twice as high in the South as in the North. Both the overall high prevalence and the geographic disparities have profound implications for the country’s healthcare system now and in the future.

Breakfast is not only the first, but also an important meal for both nutritional and familial reason. To take breakfast may be beneficial to the body and mind and to skip it may lead to consumption of snacks and to overeating by lunchtime [32]. Our finding of a negative association between regular breakfast consumption with overweight fits well with the literature. Disparities in breakfast consumption across regions may be explained by differences in cultural practices, socio-economic factors and availability of school-breakfast programs. Daily breakfast consumption (DBC) was related to all socio-demographic factors examined. We found that girls more often skipped breakfast than boys. This is in line with previous studies [33] which showed lower prevalence of DBC among girls than boys in most Italian regions [33] and in other countries [14,15,34,35]. The lower DBC in older student compared to 11 year olds is also consistent with previous studies. This age related decline in DBC could be explained by important changes that accompany adolescence including greater autonomy and independence in food choices, decreased frequency of family meals and also increased dieting, especially among girls. These findings emphasize the need for a community-level approach to prevention and health promotion which is not limited to children who are currently overweight or obese.

The geographic gradient in pediatric obesity is also seen for a wide variety of other pediatric health indicators in Italy, with the highest prevalence of most adverse outcomes found in the eight Regions of southern Italy, intermediate levels in the four central regions, and low levels in the seven regions and two autonomous Provinces of the north [22]. Bonati et al., investigating regional inequalities in child health, identified educational level, poverty and access and efficiency of health services as major determinants of this geographic gradient [36].

Our data also show a clear difference in the prevalence of overweight and obesity by age and by gender. As found in all European countries participating in the HBSC international survey, males are more likely to be overweight and obese than females, and this difference increases with increasing age [25]. The total prevalence of overweight including obesity observed in this study in children aged 11, 13 and 15 years are lower than that found in Italian children aged 8–9 years, which was 34.2% [23]. A decrease in prevalence by age is present among girls, reaching about one in ten at age 15. These trends may be due to gender differences in food choices and dietary concerns, as well as overall physical activity levels. Moreover, being thin is highly valued in Western society adolescents, in particular among females for whom it is associated with beauty. This highlights the need for a clearer focus on the environmental influences and gender differences in childhood and adolescent obesity among both researchers and policy makers.

The HBSC Italian study, based on more than 50,000 adolescents, confirms the importance of parents’ low educational level as a risk factor for overweight including obesity. The influence of this factor was found in all three age groups and was higher among girls [15]. In addition, we have found a negative association between regular breakfast consumption and overweight, as reported in other studies [7,8,37]*.* Not eating breakfast was especially high in the South and shows a higher prevalence among girls [38,39].

### Strengths and limitations

Our study has some limitations. The cross-sectional design of the study did not allow us to determine clearly the cause/effect of the relationships among the study variables; longitudinal designs might help determine the direction of these relationships. Combining overweight and obesity is another limitation of the study. Behaviors of obese students and their parents may be significantly different from those who are overweight, but not obese, even if the prevalence of obese students is very low. We did not investigate if the parent with higher education attainment was available at home. If he or she were not available at home, the impact of his/her education/knowledge on the child’s behavior may be low [40]. In addition, the analyses are not adjusted for other important nutritional factors (for example consumption of high calorific snacks or sugar-sweetened beverages) because data on snacks were not recorded in the survey and many responses to the question on sugar-sweetened beverages were missing. This may lead to the effect of breakfast consumption to be overestimated [37]. The results of our study are based on self reported data that could be subject to socially desirable reporting bias. However, students’ responses were anonymous, therefore participants had no reason to deliberately misreport the information, in particular their height or weight. According to some authors, BMI based on self-reported data can produce lower prevalence estimates of overweight and obesity than those based on actual height and weight measurements [41,42] while others have reported high accuracy for the classification of youth as obese or non-obese based on self-reported data [43,44]. The problem of the high level of non-responders (nearly 20%) was evaluated using a sensitivity analysis which showed similar results.

The main strength of the study is the use of a large and representative Italian sample which allows regional comparison and also with other countries participating in the HBSC study.

## Conclusion

In conclusion, Italy appears to have a major childhood and adolescent obesity problem that is differentially affecting the three areas of the country and is more severe among boys. A comprehensive strategy to prevent obesity in both children and adults has been a central element of the recent initiative “Guadagnare Salute” (Gaining Health) developed by the Ministry of Health [45] which is based on the World Health Organization’s initiative of the same name. The strategy calls for the involvement, not only of the health sector, but also schools, transport and agriculture, although the feasibility of the sustained attention and interventions needed through different governments is not clear. Daily breakfast consumption should be encouraged as much as possible within the context of each region and family. Increased attention to DBC is necessary during the transition from childhood to adolescence, especially in girls and young persons from disadvantaged families. Breakfast provision in schools may offer a way to overcome social inequalities in DBC and could serve to yield health benefits associated with breakfast consumption. Although more research is necessary to evaluate intermediate factors and determine which interventions are likely to be most effective to prevent childhood and adolescent obesity, rapid action is needed to avoid both present and future human and financial costs. The HBSC surveys are a useful instrument to monitor the trend of overweight and obesity among 11–15 yrs old children and the effectiveness of the interventions.

## Abbreviations

BMI: Body mass index; U: Underweight; N: Normal-weight; Ow: Overweight; O: Obesity; OwO: Overweight including obesity; DBC: Daily breakfast consumption.

## Competing interests

The authors declare that they have no competing interests.

## Authors’ contributions

GL conceptualized the study, interpreted the results, wrote manuscript and approved the final manuscript as submitted. MG drafted the initial manuscript, critically reviewed the manuscript and approved the final manuscript as submitted. AS and FC conceptualized and designed the study, interpreted study results, drafted the initial manuscript, and approved the final manuscript as submitted. PD and AP carried out the statistical analyses. AL and PN coordinated and supervised data collection, reviewed and revised the manuscript, and approved the final manuscript as submitted. All authors have read and approved the final manuscript.
